# Supplementary material for: Plasmon Effect of Ag Nanoparticles on TiO2/rGO Nanostructures for Enhanced Energy Harvesting and Environmental Remediation
Source: Nanomaterials (Basel). 2022 Dec 23;13(1):65. doi: 10.3390/nano13010065 (PMC9824111; doi:10.3390/nano13010065)
Supplement: Supplementary file 1 [file nanomaterials-13-00065-s001.zip › nanomaterials-1985004-supplementary.pdf]

nanomaterials

Supplementary Materials

Plasmon Effect of Ag Nanoparticles on TiO_2/rGO Nanostructures for Enhanced Energy Harvesting and Environmental Remediation

Seenidurai Athithya [1], Valparai Surangani Manikandan [1], Santhana Krishnan Harish [1,2], Kuppusamy Silambarasan [1], Shanmugam Gopalakrishnan [1,3], Hiroya Ikeda [2], Mani Navaneethan [1,3,*] and Jayaram Archana [1,*]

1 Functional Materials and Energy Devices Laboratory, Department of Physics and Nanotechnology, SRM Institute of Science and Technology, Kattankulathur, Chennai 603 203, India
2 Research Institute of Electronics, Shizuoka University, 3-5-1 Johoku, Naka-Ku, Hamamatsu 432-8011, Shizuoka, Japan
3 Nanotechnology Research Center (NRC), SRM Institute of Science and Technology, Kattankulathur, Chennai 603 203, India
* Correspondence: m.navaneethan@gmail.com (M.N.); jayaram.archana@gmail.com (J.A.)

Citation: Athithya, S.; Manikandan, V.S.; Harish, S.; Silambarasan, K.; Gopalakrishnan, S.; Ikeda, H.; Navaneethan, M.; Archana, J. Plasmon Effect of Ag Nanoparticles on TiO_2/rGO Nanostructures for Enhanced Energy Harvesting and Environmental Remediation. *Nanomaterials* **2023**, *13*, 65. https://doi.org/10.3390/nano13010065

Academic Editor: Muralidharan Paramsothy

Received: 22 October 2022
Revised: 10 December 2022
Accepted: 11 December 2022
Published: 23 December 2022

Copyright: © 2022 by the authors. Submitted for possible open access publication under the terms and conditions of the Creative Commons Attribution (CC BY) license (https://creativecommons.org/licenses/by/4.0/).

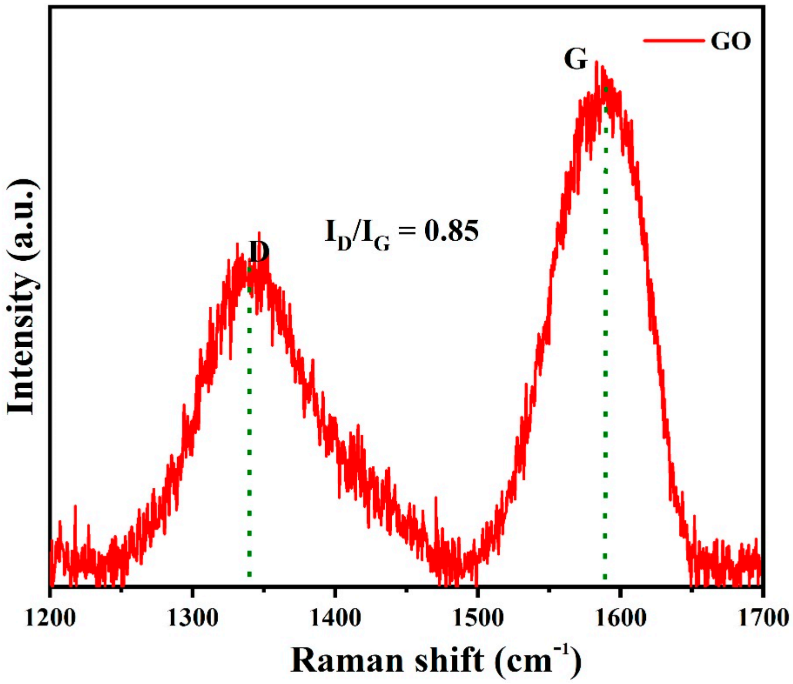

Figure S1. Raman spectra of prepared GO nanosheets.

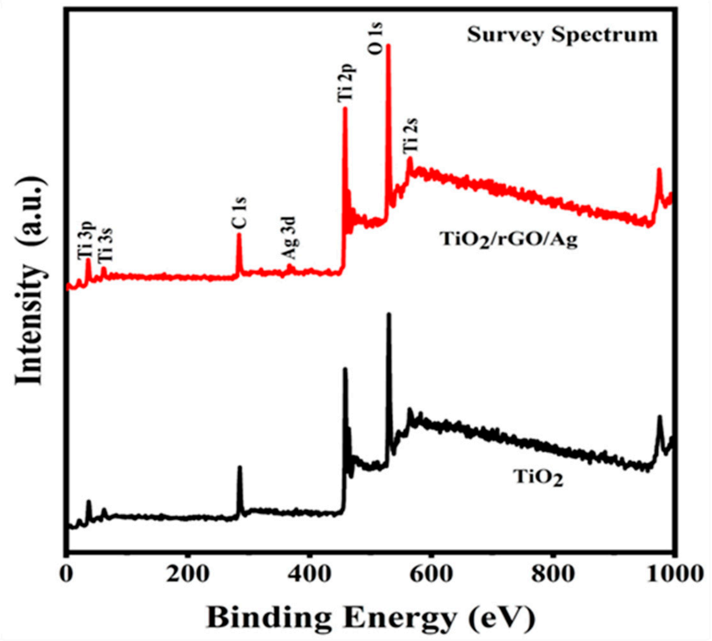

Figure S2. XPS survey spectrum of TiO_2, TiO_2/rGO and TiO_2/rGO/Ag.

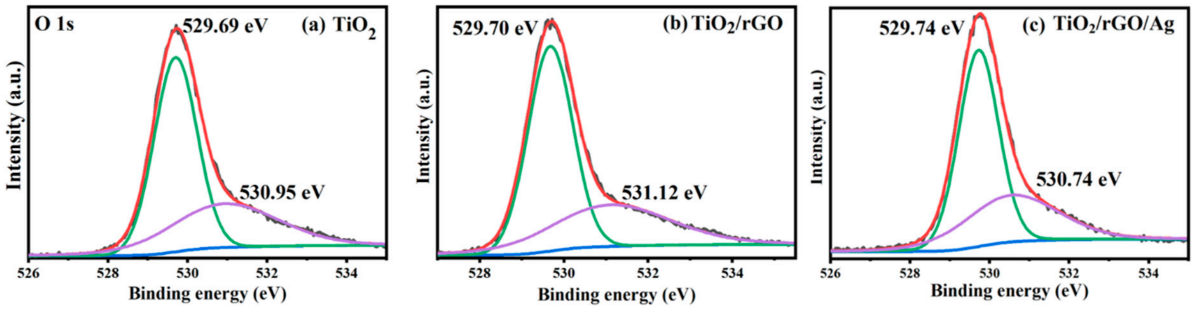

Figure S3. XPS O 1s spectra of (**a**) TiO_2 (**b**) TiO_2/rGO and (**c**) TiO_2/rGO/Ag (green line (Ti-O-Ti bond) and violet line (Ti-O-C bond)) .

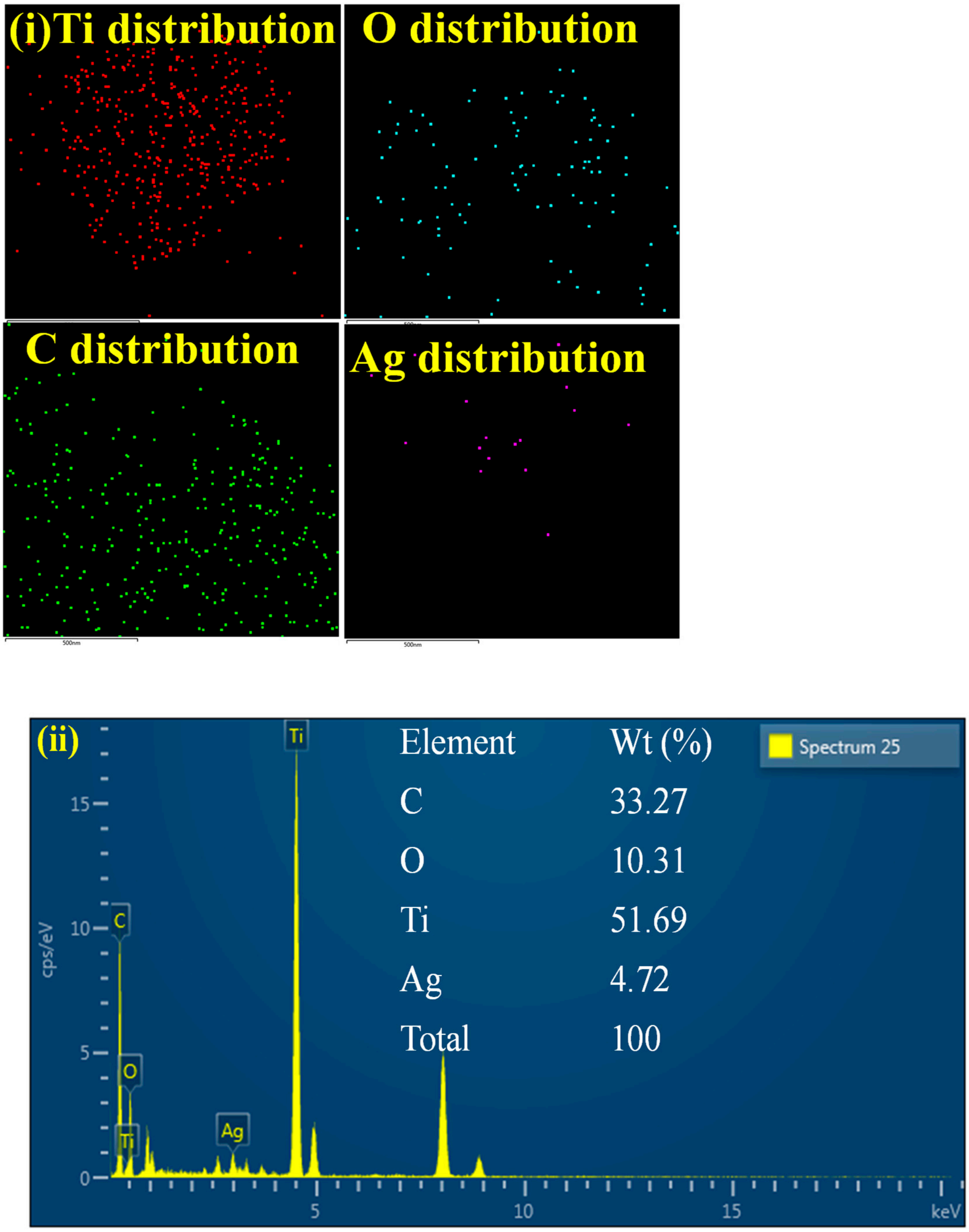

Figure S4. (**i**) STEM elemental mapping and (**ii**) EDX spectrum of TiO2/rGO/Ag hybrid ternary nanostructures.

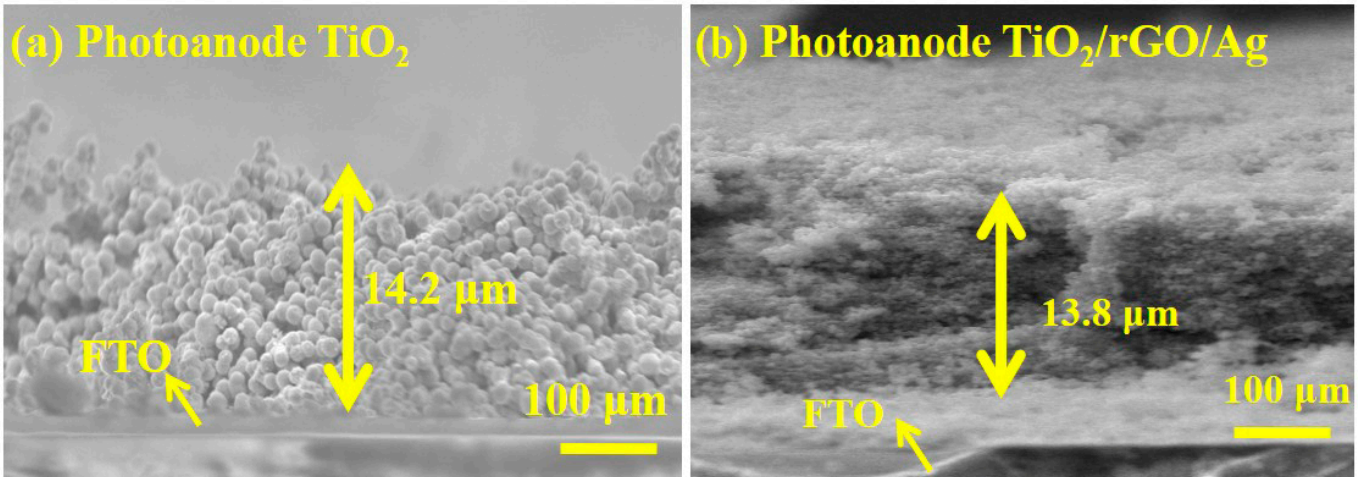

Figure S5. The HR-SEM cross section of (**a**) TiO_2 (**b**) TiO_2/rGO/Ag photoanode.

The functional group of the synthesized samples were performed by FTIR spectrum as shown in Figure S5. The strong and broad peaks are observed in range of 2800 to 3400 cm^{-1}, and it's attributed to the stretching vibration of the O-H bond (the hydroxyl groups in carboxylic acid). Besides, the other peak at 1623 cm^{-1} represents the C=O group which direct confirms the presence of GO sheet. On other hand, the hydroxyl peak has observed at 3430 cm^{-1} and 1224 cm^{-1}, respectively [1]. Then, major peak intensity of GO is reduced significantly which reveals that removal of oxygen-containing groups namely reduced grapheme oxide after the solvothermal treatment of TiO_2/rGO. Besides, the weak peak is observed at 1224 cm^{-1} due to the epoxy C-O-C stretching vibration [2]. All the peaks in FTIR study are originated from the oxygenated functional group and C=C indicating the existence of rGO in the hybrid nanostructure.

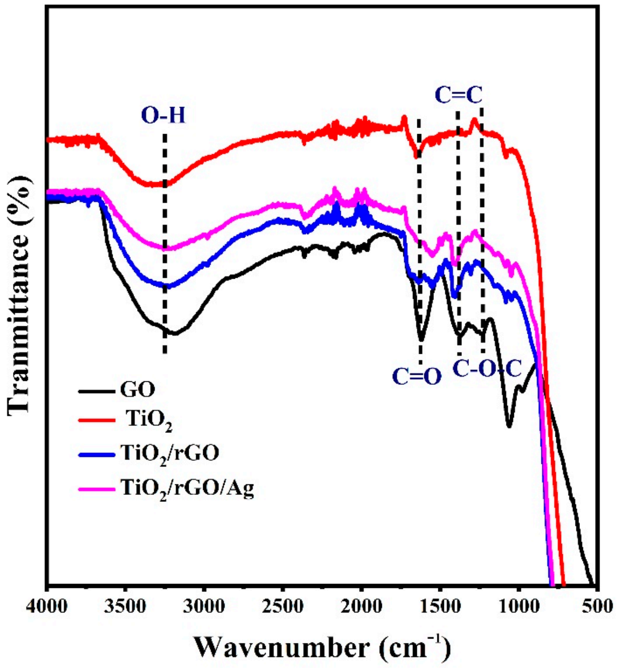

Figure S6. FTIR spectra of GO, TiO_2, TiO_2/rGO and TiO_2/rGO/Ag.

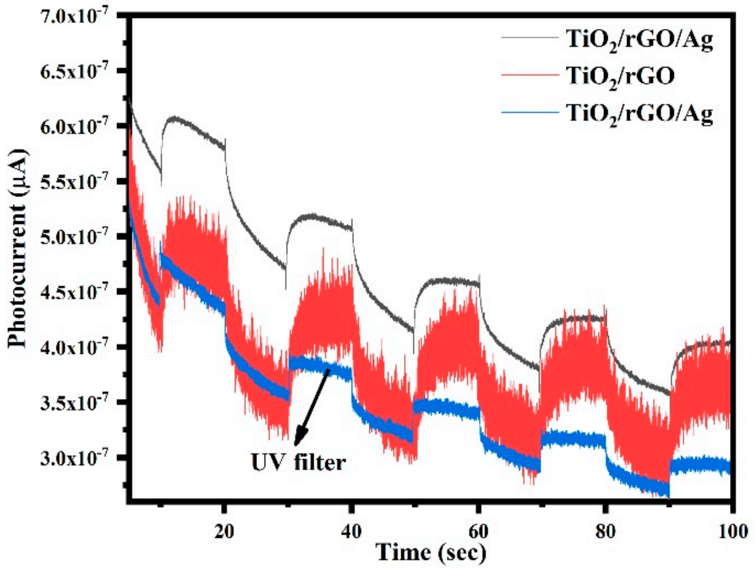

Figure S7. Transient photocurrent spectra of TiO2/rGO and TiO2/rGO/Ag (under with and without UV filter).

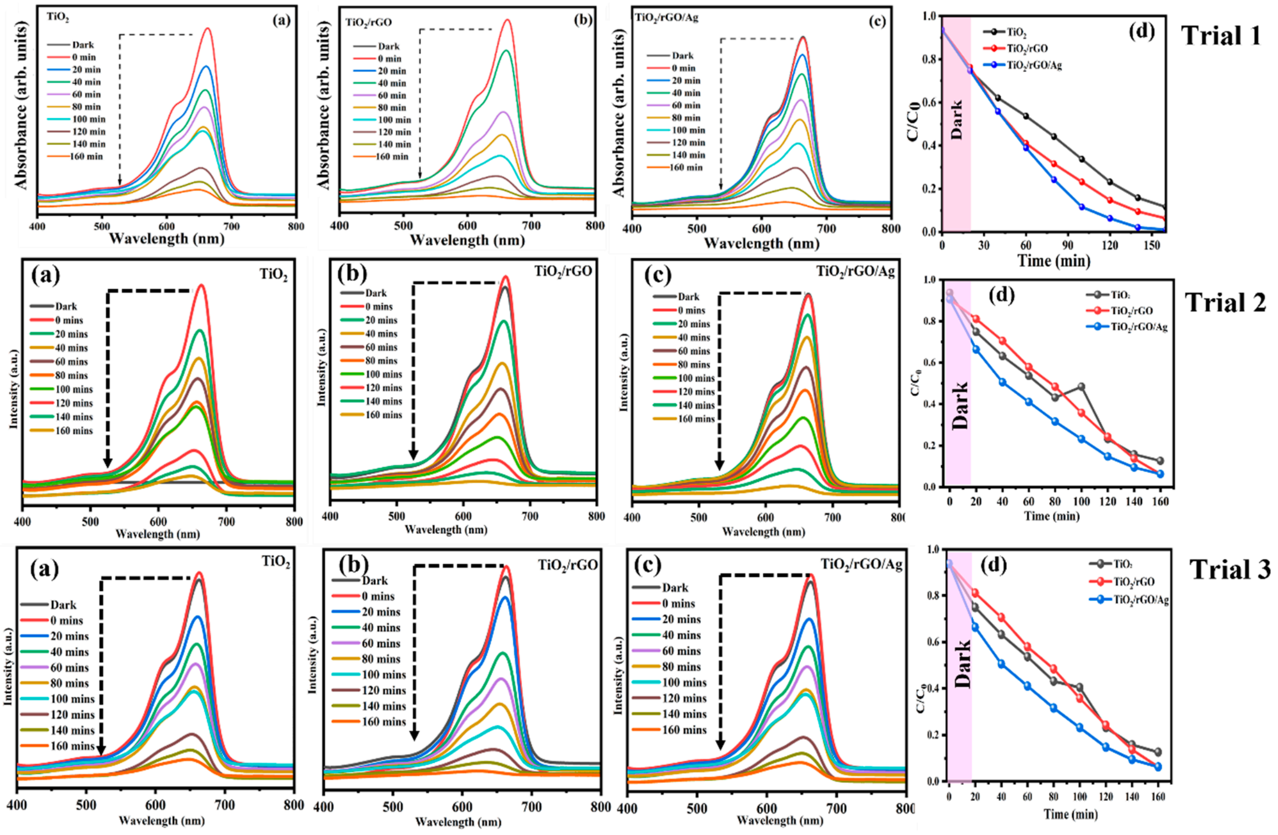

Figure S8. (a-c) Three different trials of UV absorption spectra of MB degradation with different interval time under natural sunlight. (**d**) Plots of Ln (C/C0) as a function of time (min) towards the photo degradation of MB.

References

1. Zhang, H.; Wang, X.; Li, N.; Xia, J.; Meng, Q.; Ding, J.; Lu, J. Synthesis and characterization of TiO_2/graphene oxide nanocomposites for photoreduction of heavy metal ions in reverse osmosis concentrate. *RSC Adv.* **2018**, *8*, 34241–34251, https://doi.org/10.1039/c8ra06681g.
2. Qiu, B.; Zhou, Y.; Ma, Y.; Yang, X.; Sheng, W.; Xing, M.; Zhang, J. Facile synthesis of the Ti3+ self-doped TiO2-graphene nanosheet composites with enhanced photocatalysis. *Sci. Rep.* **2015**, *5*, 8591–8591, https://doi.org/10.1038/srep08591.

Disclaimer/Publisher's Note: The statements, opinions and data contained in all publications are solely those of the individual author(s) and contributor(s) and not of MDPI and/or the editor(s). MDPI and/or the editor(s) disclaim responsibility for any injury to people or property resulting from any ideas, methods, instructions or products referred to in the content.
